# Evidence-based information needs of public health workers: a systematized review

**DOI:** 10.5195/jmla.2017.109

**Published:** 2017-01

**Authors:** Jill Barr-Walker

## Abstract

**Objective:**

This study assessed public health workers’ evidence-based information needs, based on a review of the literature using a systematic search strategy. This study is based on a thesis project conducted as part of the author’s master’s in public health coursework and is considered a systematized review.

**Methods:**

Four databases were searched for English-language articles published between 2005 and 2015: PubMed, Web of Science, Library Literature & Information Science Index, and Library, Information Science & Technology Abstracts (LISTA). Studies were excluded if there was no primary data collection, the population in the study was not identified as public health workers, “information” was not defined according to specific criteria, or evidence-based information and public health workers were not the major focus. Studies included in the final analysis underwent data extraction, critical appraisal using CASP and STROBE checklists, and thematic analysis.

**Results:**

Thirty-three research studies were identified in the search, including twenty-one using quantitative methods and twelve using qualitative methods. Critical appraisal revealed many potential biases, particularly in the validity of research. Thematic analysis revealed five common themes: (1) definition of information needs, (2) current information-seeking behavior and use, (3) definition of evidence-based information, (4) barriers to information needs, and (5) public health–specific issues.

**Conclusions:**

Recommendations are given for how librarians can increase the use of evidence-based information in public health research, practice, and policy making. Further research using rigorous methodologies and transparent reporting practices in a wider variety of settings is needed to further evaluate public health workers’ information needs.

## INTRODUCTION

Evidence-based information in public health (EBPH) is an emerging topic in the field of public health. There are many important components to EBPH, but this review will focus on the aspect of EBPH that is defined as making decisions on the basis of the best available scientific evidence [1]. Guidelines in the literature describe the use of EBPH and stress the importance of this practice [2–4]. EBPH is often used to create interventions, with a general recognition that this approach is essential to changing public health outcomes [5]. Similarly, there is demand for public health policies to be based on existing evidence [6], and the principles of EBPH are increasingly being taught in public health departments [5, 7, 8]. Despite the belief that these concepts are important in public health, the use of evidence-based information remains underutilized in practice, and research plays a limited role in the formulation of policy and interventions in public health [6]. There are many possible barriers to the use of evidence-based information in public health, such as a lack of knowledge and skills regarding EBPH, lack of communication of evidence-based research findings to policy makers, lack of an organizational culture that supports EBPH, and lack of funding for EBPH resources [9, 10].

Librarians have been on the forefront of the evidence-based information movement by providing instruction and support to researchers. Librarians who serve public health workers, including medical and academic librarians, have a unique opportunity to ensure that this population utilizes evidence in their research and practice. Despite the variety of settings that employ public health workers, research on this topic has traditionally focused on those working in clinical settings [11, 12] and government departments [13, 14]. Few studies have surveyed public health workers in other occupational contexts, and currently no systematic reviews involve the information needs of public health workers in academia or private organizations.

Given the importance of evidence-based information to the fields of public health and library and information science, the knowledge, access, and use of this information by public health workers is a topic worthy of study. Although literature reviews on this topic exist [15, 16], none have employed systematic search strategies or critical appraisal. The main aim of this study is to assess information needs of public health workers based on a review of the literature using a systematic search strategy.

## METHODS

### Explanation of methods

This project was originally completed as a master’s thesis as part of the author’s master’s in public health coursework at the London School of Hygiene and Tropical Medicine. Because the author conducted the review as a solo project, it should not be considered a systematic review, but rather a systematized review [17]. The potential biases that may result from this methodology are discussed in the limitations section.

### Search strategy

Searches were carried out to address the question: what are the information needs of public health workers? Because the search question includes concepts from library and information science and public health, the following four databases were searched: PubMed, Web of Science, Library Literature & Information Science Index, and Library, Information Science & Technology Abstracts (LISTA). Gray literature was not included in the search. The field of library and information science has a well-established network of journals, and research in this field is published and disseminated through traditional channels. While public health researchers often create gray literature, such as unpublished reports, this method is not used widely in librarianship. Since the research question contains aspects related to this field as well as public health, the most relevant studies were expected to appear in databases that contained published literature.

The search was undertaken on April 1, 2015. Search terms included information, public health, librarian, evidence-based policy, and other relevant keywords based on the concepts of information needs and EBPH. A complete list of searches that were conducted can be found in supplemental Appendix A. Initial search terms were broad, and, upon review of the results, searches were narrowed to include greater numbers of relevant articles and increase the specificity of results. For example, a search in Web of Science for “public health” AND “information” yielded over 11,000 results, many of which were not relevant to the research question. Refining the search term to “public health information” yielded a result of 181 articles, the majority of which were relevant. Citation checking in Web of Science was used, and hand-searching, including reference searching and key author searching, was also utilized.

Date and language limits were placed on the search: only English-language articles and those published between 2005 and 2015 were included. Database searching yielded 1,615 articles. After the removal of 405 duplicates, 1,210 articles were included in title and abstract screening. During the title and abstract screening, 1,078 articles were excluded, leaving 132 studies to evaluate during full-text screening.

### Exclusion criteria

To maintain the focus on public health workers and information needs, exclusion criteria were used. Studies were excluded if there was no primary data collection, the population in the study was not identified as public health workers, “information” was not defined as below, or the major focus of the study was not about evidence-based information or public health workers.

To define exclusion criteria, essential aspects of the research question were defined. A public health worker was defined as “a person educated in public health or a related discipline who is employed to improve health through a population focus” [18]. Information was defined as “any stimulus that reduces uncertainty in a decision-making process,” and an information need was defined as “the recognition of what information can reduce this uncertainty as well as unrecognized or potential information needs” [3]. All definitions were taken from a well-designed study of public health workers’ information needs that Revere et al. conducted [16]. Exclusion of studies without primary data collection was necessary to limit the scope of this review to original research.

**Figure 1:**
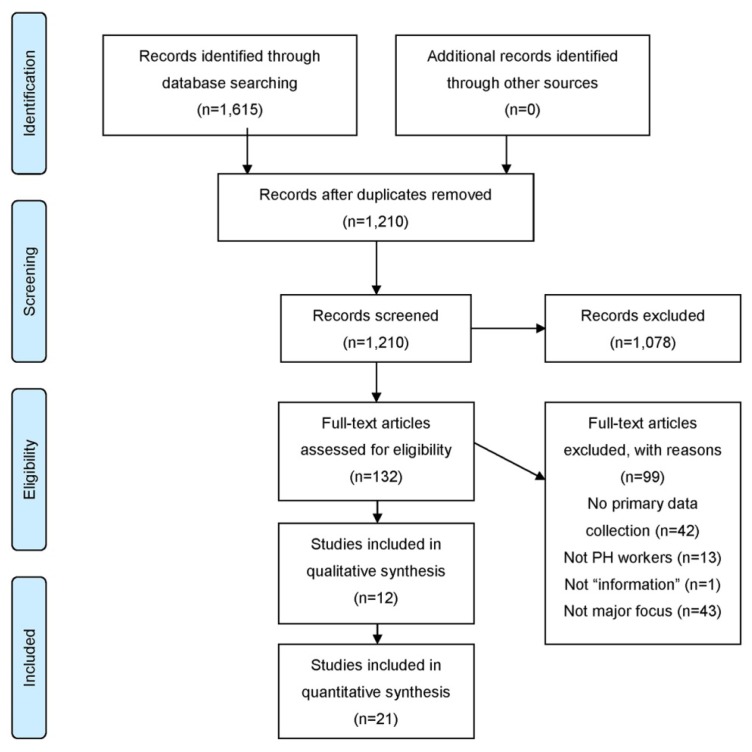
PRISMA flow diagram [53]

During full-text screening, ninety-nine studies were excluded. A list of reasons for exclusion is included in Figure 1. Thirty-three studies were included in the final analysis.

### Data extraction

Data were extracted from each study using the criteria outlined in the Critical Appraisal Skills Programme (CASP) checklist for qualitative studies [19] and the STrengthening the Reporting of OBservational studies in Epidemiology (STROBE) checklist for quantitative studies [20]. A form was created based on these checklists that asked fourteen questions of each qualitative study and twenty-two questions of each quantitative study. Extracted data included the type of study population, sample size, outcome measures, and potential sources of bias. The data extraction checklists can be found in supplemental Appendixes B and C.

### Critical appraisal

Critical appraisal is the process of systematically examining research to judge its trustworthiness, value, and relevance in a particular context [21]. It is used to evaluate the quality of the studies examined, detect biases, and assess and discuss the validity, relevance, and usefulness of research evidence [22]. Data from each of the studies was critically appraised using the CASP checklist for qualitative studies [19] and the STROBE checklist for quantitative studies [20]. The questions used in the critical appraisal checklists can be found in Appendixes B and C.

### Data synthesis

A thematic synthesis was applied to the studies, whereby the findings were examined for analytical themes and compared across studies [23]. The findings were reviewed for key concepts and recurring themes, gaps in the current evidence base, and potential areas for further research.

### Ethical considerations

Ethical approval was obtained from the London School of Hygiene and Tropical Medicine on February 16, 2015.

## RESULTS

### Critical appraisal

#### Descriptive data

Twenty-one quantitative studies and 12 qualitative studies were included in this review. A summary of study characteristics can be found in Table 1. All quantitative studies used surveys, while 10 qualitative studies used semi-structured interviews, 6 used focus groups or observational methods, and 2 used surveys. One study used a randomized controlled trial study design [24]. Sample sizes ranged from 6 to 904. The median sample size for quantitative studies was 134, and the median for qualitative studies was 35. Many researchers used convenience samples, often consisting of academic or government employees of departments where the study was conducted [25–28]. There were several types of participants: health department employees, academic faculty, and clinical public health workers were the most common. On average, participants were young, with typically less than 5 years of total experience or time at their current jobs. In surveys of health department directors, the amount of experience was considerably higher.

**Table 1:** Descriptive summary of studies

Author	Year	Type of study	Setting	Participant group	No. of participants	Themes
Adily & Ward [26]	2005	Quantitative	Australia	LHD	76	Evidence, barriers
Alvarez et al. [30]	2013	Qualitative	Brazil	PHRF	6	Evidence, barriers
Andualem et al. [37]	2013	Quantitative	Ethiopia	PHW	339	Info needs, info use, barriers
Brownson et al. [2]	2014	Quantitative	US: nationwide	DHD	517	Evidence
Campbell et al. [31]	2009	Qualitative	Australia	PM, PHRF	79	Info needs, info use, barriers
Charbonneau et al. [32]	2007	Quantitative	US: Michigan	LHD, PHN, PHW	105	Info needs
Cilenti et al. [39]	2012	Qualitative	US: two unspecified regions	PHF, DHD	22	Barriers
De Groote et al. [33]	2014	Quantitative	US: Illinois	PHRF	198	Info needs, info use, barriers
Dodson et al. [50]	2010	Qualitative	US: nationwide	DHD	469	Evidence, barriers
Eldredge et al. [25]	2008	Quantitative	US: New Mexico	SHD	60	Info use
Harris et al. [34]	2014	Quantitative	US: nationwide	SHD	904	Info needs, info use, barriers
Higgins et al. [43]	2011	Qualitative	Canada	LHD	21	Info needs, barriers
Jacobs et al. [10]	2010	Quantitative	US: nationwide	DHD	447	Evidence, barriers
Jacobs et al. [44]	2012	Quantitative	US: Mississippi and Kansas	SHD, LHD	262	Info needs, evidence, barriers
Kapadia-Kundu et al.	2012	Qualitative	India	PHW, CPHW	55	Evidence, barriers
[40]						
LaPelle et al. [48]	2006	Qualitative	US: Massachusetts	SHD	19	Evidence, barriers, public health–specific
Larsen et al. [35]	2012	Quantitative	Denmark	DHD	98	Info use, evidence, barriers
Lê [13]	2013	Quantitative	Canada		18	Info needs, info use, barriers
Lê [27]	2014	Quantitative	Canada	MPH	38	Info needs, info use
Léon et al. [45]	2013	Quantitative	Canada	LIB	14	Info needs, info use
Maylahn et al. [36]	2008	Quantitative	US: New York	LHD	166	Info needs, info use, evidence, barriers
Merrill et al. [41]	2007	Qualitative	US		137	Info needs, info use, barriers
Mortensen et al. [46]	2013	Quantitative	US: Western region	PHW	157	Info needs, info use
Peirson et al. [51]	2012	Qualitative	Canada	LHD	70	Evidence, barriers, public health-specific
Raj et al. [42]	2015	Quantitative	India	PHW, CPHW	100	Info needs, info use, barriers
Rutland and Smith [11]	2010	Qualitative	England	CPHW	8	Info needs, info use
Sosnowy et al. [47]	2013	Qualitative	US: New York	LHD	47	Info needs, info use
Turner et al. [12]	2008	Qualitative	US: Oregon	PHN	17	Info needs, info use, evidence, barriers
Turner et al. [28]	2009	Quantitative	US: Northwestern region	DHD, LHD	134	Info needs, info use
Twose et al. [14]	2008	Quantitative	US: Maryland	LHD	18	Info needs
Wallis [29]	2006	Quantitative	US: Illinois	PHF	45	Info needs, info use, barriers
Yousefi-Nooraie et al. [49]	2012	Quantitative	Canada	LHD	196	Info use
Zardo and Collie [23]	2015	Quantitative	Canada	PHW	372	Info use, evidence

Abbreviation key

Participant groups: CPHW=clinical public health workers (excluding nurses), DHD=directors of health departments, LIB=library employees, LHD=local health department employees (excluding directors), MPH=master’s of public health students, PHF=public health academic faculty or researchers, PHN=public health nurses, PHW=public health workers not included in any of the other categories, PM=policy makers, SHD=state health department employees (excluding directors).

Eighteen studies were conducted in the United States, with eleven occurring in one location and seven taking place either nationwide or in multiple states or regions. Seven studies were conducted in Canada, and four were conducted in Europe or Australia. Four studies were conducted in non-Western countries: India, Brazil, and Ethiopia.

#### Quality of evidence

There were several recurrent methodological issues in the studies. The most frequently occurring issues were low generalizability, selection bias related to response rate, small sample size, selection bias related to sampling, no data analysis, self-reporting used as a data collection tool, poor survey design, interviewer bias, and the lack of author acknowledgement of bias. A list of critical appraisal issues observed in the studies can be found in Table 2.

**Table 2:** Critical appraisal issues that occurred in the studies

Critical appraisal issue observed	Number of studies
Low generalizability	20
Selection bias related to response rate	13
Small sample size	13
Author did not address bias	10
No data analysis	9
Selection bias related to sampling	9
Self-report	7
Poor survey design	4
Interviewer bias	2

Note: Several studies displayed more than 1 critical appraisal issue; thus, the total amount here does not add up to the total number of studies (33).

The most common issue for critical appraisal that was observed across studies was a lack of information provided, often about sampling strategy, response rates, and data analysis. For this reason, it was not possible to complete the entire CASP or STROBE checklist for most studies.

Eight quantitative and 2 qualitative studies did not use data analysis [14, 26, 29, 30–36]. Most qualitative studies used thematic analysis and coding, but 40% of quantitative studies did not use any statistical analysis methods. Quantitative studies most often used descriptive statistics and tested bivariate relationships using chi-square or *t*-test methods. Several studies calculated odds ratios [10, 37, 38].

Many survey studies had low response rates, and authors infrequently addressed possible reasons for this. Overall, response rates ranged from 25%–100%, with a median of 65%. In 10 studies, the authors did not acknowledge or address possible biases in their research [25, 26, 29, 31, 37, 39, 40–43].

### Data synthesis

Five themes emerged during thematic analysis: definition of information needs, current information-seeking behavior and use, definition of evidence-based information, barriers to information needs, and public health-specific issues. Results of critical appraisal did not affect this analysis; all studies were weighted equally regardless of potential biases.

#### Theme 1: Definition of information needs

Twenty studies were devoted to examining the information needs of public health workers [11–14, 26–28, 30–33, 35–37, 42, 44–47]. Participants self-reported these needs, usually from a list of items in a survey. Information needs were defined as the types of information that public health workers need in their daily work. Several studies reported that public health workers need statistics, government reports and guidelines, and journal articles [11, 27, 31, 40, 42, 46, 48]. Staying current with the latest public health research, finding data, and finding materials for grant-writing were other areas of need [11, 13, 14, 32, 33, 49]. Participants frequently expressed the need for librarians but were often uncertain about the services that librarians could provide [11, 13, 26, 28, 29, 46]. Information needs often differed according to the roles and positions of participants [12, 40]. Local health department employees, for example, were more interested in practical knowledge, while state health departments had an increased need for guidelines and program planning [40].

#### Theme 2: Current information-seeking behavior and use

Twenty studies discussed current information-seeking behavior and the use of information [11–13, 24, 26–28, 30, 32–37, 41–43, 46–48]. Data were collected by asking which information sources public health workers used in their work, usually in the form of a list. The most commonly selected information source was PubMed/MEDLINE, followed by Google and the Centers for Disease Control and Prevention (CDC) website [12, 13, 26, 27, 32, 49]. In one study, few participants were aware of or used any databases besides PubMed [32]. Journal articles were identified as one of the main sources of information across participant types [13, 26, 28, 32, 33, 35, 46, 49]. Many public health workers used colleagues as a source of information, often more frequently than they used online databases or librarians [12, 13, 27, 37, 42, 44, 48, 49]. Librarians were not a heavily used resource, and there was a prevailing lack of knowledge about the role of librarians and the services they could provide [11, 13, 26, 28, 29, 46].

Discrepancies between information needs and information use among public health workers were observed. Information needs as defined by public health workers included journal articles, peer-reviewed information, and help finding data. Reports of public health workers’ resource use differed from these findings: although use of PubMed was common, websites like Google and CDC were consistently rated as top information sources, while online databases containing peer-reviewed journal articles were rated among the lowest used [12, 13, 26, 27, 32, 49]. Although highly rated for information needs, librarians were underutilized, with many participants expressing uncertainty about their ability to access librarian services at their workplaces [11, 13, 26, 28, 29, 46]. One survey study found that while collaboration with other researchers was rated as very important, file sharing and collaborative data management programs like EndNote were utilized by less than 10% of participants [32].

#### Theme 3: Definition of evidence-based information

Thirteen studies examined EBPH information [2, 10, 12, 25, 29, 34, 36, 40, 43, 45, 50, 51]. These studies surveyed health department employees, directors, and policy makers to learn about the use of evidence-based information in decision making (EBDM). Across studies, EBDM was consistently defined as an important tool that should be used by public health workers. In practice, however, public health workers did not use EBDM regularly [36, 38, 47]. Public health workers’ lack of knowledge about EBDM and the concept of evidence was demonstrated in several studies [25, 34, 36, 47, 50]. One study asked participants to define evidence and received diverse responses that represented a range of knowledge about this topic [44]. Those with less education in public health and epidemiology felt the least confident in using EBDM and reported the lowest numbers of finding and using evidence in their work [10, 36, 50]. Overall, public health workers were interested in increased training for EBDM, and this finding held true across workplace type and education level [25, 36, 39, 45, 47, 50, 51].

#### Theme 4: Barriers to information

Eighteen studies surveyed participants about internal and external barriers to finding, accessing, and using information in their work. Internal barriers included lack of time, funding, training, staff, equipment, and subscriptions to journals [10, 11, 36–38, 42, 47, 49–51]. Public health workers reported that training was needed on how to find, access, evaluate, and synthesize information [12, 13, 25, 27, 32, 36, 45, 47, 49, 50]. Training was also needed on the processes involved in EBDM, including an explanation of what evidence consists of, how to find it, and how to use it in real-world situations [12, 13, 25, 27, 32, 36, 45, 47, 49, 50]. An inability to access information because of technical issues or lack of funding that led to the unavailability of full-text articles was also reported [10, 11, 36–38, 42, 47, 49–51]. The political climate, including local agendas that did not prioritize EBDM, was a common external barrier [10, 30, 34, 38, 39, 45, 50]. The need for organizational change and managerial support was cited as another significant barrier to using EBDM as this lack of support influenced internal factors like funding, training, and time [10, 11, 38, 45, 47, 50, 51]. Low-income countries reported the unavailability of computer equipment and lack of computer literacy as considerable barriers to using evidence-based information [37, 42]. One older study in the United States also mentioned these factors [12].

#### Theme 5: Public health–specific issues

The final theme addressed public health–specific issues related to finding and using evidence-based information. Public health is an interdisciplinary field, and public health workers must search multiple subject databases, many of which might not reflect new trends in the field [44, 50]. In several of the studies in this review, gray literature, statistics, and government guidelines were cited as important public health sources that public health workers frequently used [11, 12, 27, 31, 40, 42, 46, 48, 49], but traditional online databases do not contain these materials.

Public health workers in several studies reported that using evidence could conflict with their mandate of community empowerment if community members identified different priorities than those recommended in evidence-based research [49, 51]. In some research settings, especially those involving underserved populations, there was a lack of evidence for public health topics, and the lack of transferability could preclude use of an evidence base for these populations [25, 44, 47, 50].

## DISCUSSION

Public health workers see EBPH as a high-priority initiative [25, 36, 39, 45, 47, 50, 51]. However, there is a discrepancy between the stated importance of this process and its actual practice. It is apparent that public health workers are uncertain about some of the very basic aspects of this concept, including things like the definition of evidence, where to find it, and how to use it [36, 38, 47, 51]. Interestingly, few studies about information needs of public health workers considered these needs in relation to EBDM, although this initiative is increasingly important in public health departments. A consistent message that public health workers presented was their desire for training in EBDM and finding and using evidence in their work [25, 32, 36, 44, 45, 47, 51]. Although they expressed information needs covering a wide range of sources, very few public health workers were aware of the full range of services that libraries provide, including access to peer-reviewed articles and gray literature. Importantly, there was a distinct disconnect in the identification of needs and the awareness that librarians could assist with these needs. For example, many public health workers expressed the need to learn more about identifying and evaluating relevant articles when dealing with large amounts of information, but none acknowledged that this was a service that librarians could provide [12, 13, 27, 30, 36, 44, 45, 47, 49].

Each of these findings presents an opportunity for librarians to showcase their skills in finding, accessing, and using information. Librarians can use their evidence-based information skills to teach public health workers about EBDM. Even if librarians do not have advanced degrees in public health, their knowledge of the scientific information life cycle, including the production and use of evidence, can be leveraged in instruction. Librarians should also be aware of public health–specific research needs, including the importance of unpublished material like gray literature and statistics. While teaching public health workers to use evidence-based information, librarians should acknowledge the dearth of research on underserved populations in the public health evidence base; further research in this area should be encouraged and prioritized.

Some of the discrepancy between reported information needs and information use among public health workers may be due to a lack of awareness of library collections and services. Teaching public health workers about the importance of evaluating information found online may encourage them to use peer-reviewed journal databases rather than unauthoritative Internet sources. Raising awareness about the services that a librarian can provide, such as creating search strategies and setting up data management plans, can encourage public health workers to utilize librarians rather than asking other colleagues for help. Librarians should work closely with stakeholders, including directors of public health departments and public health faculty, to ensure buy-in and participation from the top down of an organization; it is essential that librarians and public health decision makers work together to increase the awareness of evidence-based information resources to be used in public health work.

This review replicates the findings of previous studies that found access, funding, and time to be major barriers to using evidence-based information [15, 16]. Additionally, several studies in this review highlighted external factors like political climate, organizational culture, and funding mandates [10, 30, 39, 45, 47, 50]. Funding mandates that require evidence-based information to be used in research can potentially increase organizational support for EBDM and should be supported.

Critical appraisal identified many potential biases in the quality of studies. Many studies used small samples or convenience samples to evaluate services to local populations [26, 28, 32]. The use of these techniques, while common in qualitative studies, can potentially limit generalizability of studies in their applicability to different settings. Public health directors were a potentially overrepresented group, as they were surveyed exclusively in several studies [10, 27, 30, 38, 47, 50]. Directors are often responsible for new initiatives like EBDM, but questioning them exclusively may lead to overrepresentation of their views in relation to other public health workers.

Potential selection bias can also occur because of issues related to sampling and response rate across studies. In studies that used nationwide surveys, the sample was often drawn from member associations like the National Association of Chronic Disease Directors, a group that is very narrow in scope [10, 50]. A methodological issue specific to survey studies was the lack of attention paid to participant flow. In several cases, respondents did not complete all questions or there was a high dropout rate, but this was not reflected in the analysis of results [24, 48].

Self-report, a commonly used data collection method, relies on participants’ memory or perceived needs. Additional measurements that assessed actual use of databases, for example, would have been a welcome addition to address potential self-reporting concerns. Importantly, author-addressed bias was an often overlooked evaluation. Studies that did address bias frequently cited self-reporting [10, 35, 38, 45, 47, 48], small sample size [12, 13, 30, 32, 34, 38, 44, 45], lack of generalizability because of a limited sample [11–14, 27, 28, 32, 34, 46, 47, 49, 51], and low response rates [10, 24, 36, 45, 50].

The most striking finding of the critical appraisal analysis is the fact that many studies omitted information about data analysis, response rates, sampling strategies, and potential biases. Ten out of 33 studies, including 40% of qualitative and 16% of quantitative studies, did not use any type of data analysis to evaluate their results. The lack of data analysis points to a possible gap in knowledge of the use of statistical data analysis methods, especially among librarian researchers. Even performing a simple bivariate analysis illustrates relationships between the results in a way that presenting raw data does not. Perhaps even more striking is the lack of information presented about sampling strategy, response rates, and data analysis in many of the studies. One-third of the studies did not address potential bias in their work, and many did not include information about how participants were selected or data analysis was conducted. These findings speak to a need for transparency in presenting research methods and education in the use of rigorous methodologies. Librarians and public health workers alike may require training in research methods to understand the importance of reducing bias, improving validity, and increasing transparency in reporting research findings.

### Limitations

There are several potential problems with having a single author on a systematic review. This practice is not recommended by the Institute of Medicine because of the potential for bias in selection, final screening, coding, and analysis [52]. For this reason, the current study should be categorized as a systematized review rather than a traditional systematic review, and such potential biases should be considered limitations of the current study [17].

The search strategy used the broad term “public health” and did not specify specialties in this field such as epidemiology or health promotion, which might have excluded relevant subject-specific studies. Four databases, two that included public health literature and two that included library and information science literature, were used in the search, which might have limited the results from the public health field. Gray literature and non-English publications were excluded, which might have further limited the results. Older studies, as far back as 2005, were included, which might have influenced some of the themes, including technological access as a barrier to using evidenced-based information [27]. Rigorous coding and thematic analysis methods, including line-by-line coding, were not used, and there were not multiple coders. This may have led to bias in the identification of themes.

As noted in “Critical Appraisal,” many of the studies had methodological issues. Therefore, definitive conclusions about themes or patterns cannot be made in regard to the topic of information needs and use of evidence among public health workers. Future reviews of the evidence should take the quality of studies into account when analyzing this body of work.

## CONCLUSION

Librarians can help improve understanding and use of EBPH by raising awareness of evidence-based resources among public health workers. Partnerships between librarians and public health decision makers can lead to increased use of EBPH in research in all types of public health work environments. Future research on public health workers’ information needs should focus on the use of evidence in decision making and practice, and such research should attempt to address potential biases by using rigorous methodologies and transparency in reporting results.

## SUPPLEMENTAL FILES

Appendix A List of search terms and results for each database

Appendix B Critical Appraisal Skills Programme (CASP) qualitative checklist for qualitative studies

Appendix C STrengthening the Reporting of OBservational studies in Epidemiology (STROBE) checklist for cross-sectional studies
