# Supplementary material for: Evidence-based information needs of public health workers: a systematized review
Source: J Med Libr Assoc. 2017 Jan;105(1):69–79. doi: 10.5195/jmla.2017.109 (PMC5234453; doi:10.5195/jmla.2017.109)
Supplement: Appendix A [file jmla_jan17_barr_appa.pdf]

JMLA Journal of the Medical Library Association

SC
Supplemental content to
J Med Libr Assoc. Jan;105(1): dx.doi.org/10.5195/jmla.2017.109
www.mlanet.org
© Barr-Walker 2017

Evidence-based information needs of public health workers: a systematized review

Jill Barr-Walker, MPH, MS

APPENDIX A

List of search terms and results for each database

Database searched	Search terms used	Number of results
PubMed	"public health" AND "information needs"	196
PubMed	"public health" AND "information access"	28
PubMed	"public health" AND librarian	48
PubMed	"evidence based public health"	193
PubMed	("Public Health"[tiab]) AND "evidence based" [ti] AND policy[tiab]	87
PubMed	"information seeking" AND "public health"	166
Web of Science	"public health information"	181
Web of Science	"public health" AND librarian	19
Web of Science	"public health" AND "information needs"	107
Web of Science	"public health" AND "information access"	23
Web of Science	"public health" AND "knowledge management"	45
Web of Science	"evidence based public health"	174
Library Literature & Information Science Index	"public health" AND information needs	22
Library Literature & Information Science Index	"public health" AND information access	80
Library Literature & Information Science Index	"public health" AND evidence based	14
Library, Information Science & Technology Abstracts (LISTA)	"public health"" AND "information needs"	55
LISTA	"public health" AND "information access"	23
LISTA	" public health" AND evidence based	154
All databases	Total results	1,615
