# Supplementary material for: Evidence-based information needs of public health workers: a systematized review
Source: J Med Libr Assoc. 2017 Jan;105(1):69–79. doi: 10.5195/jmla.2017.109 (PMC5234453; doi:10.5195/jmla.2017.109)
Supplement: Appendix B [file jmla_jan17_barr_appb.pdf]

JMLA Journal of the Medical Library Association

SC
Supplemental content to
J Med Libr Assoc. Jan;105(1): dx.doi.org/10.5195/jmla.2017.109
www.mlanet.org
© Barr-Walker 2017

Evidence-based information needs of public health workers: a systematized review

Jill Barr-Walker, MPH, MS

APPENDIX B

Critical Appraisal Skills Programme (CASP) qualitative checklist for qualitative studies

From http://www.casp-uk.net/#!casp-tools-checklists/c18f8.

1. Was there a clear statement of the aims of the research?
2. Is a qualitative methodology appropriate?
3. Was the research design appropriate to address the aims of the research?
4. Was the recruitment strategy appropriate to the aims of the research?
5. Were the data collected in a way that addressed the research issue?
6. Has the relationship between researcher and participants been adequately considered?
7. Have ethical issues been taken into consideration?
8. Was the data analysis sufficiently rigorous?
9. Is there a clear statement of findings?
10. How valuable is the research?
